# Evaluation of the Accuracy of Digital Models Generated Using Photogrammetry

**DOI:** 10.7759/cureus.75448

**Published:** 2024-12-10

**Authors:** Somil Chaudhary, Sandeep Kumar, Rajnish Aggarwal, Japjee Uppal, Kanika Yadav, Bhavna Thoidingjam, Kulashekar Reddy Nandalur, Vinod Bandela

**Affiliations:** 1 Department of Prosthodontics, Surendera Dental College and Research Institute, Sri Ganganagar, IND; 2 Department of Prosthetic Dental Sciences, College of Dentistry, Jazan University, Jazan, SAU; 3 Department of Prosthetic Dental Sciences, College of Dentistry, Jouf University, Sakaka, SAU

**Keywords:** accuracy of casts, digital dental models, digital dentistry, photogrammetry, software

## Abstract

Introduction: In contemporary clinical settings, three-dimensional (3D) models have become an integral component of daily practice. Photogrammetry, a novel method in clinical practice, enables the creation of precise 3D models from small objects while maintaining their original shape and size.

Aim: To evaluate the accuracy and reliability of digital models (DM) generated using photogrammetry techniques compared to traditional gypsum models (GM) and to investigate the feasibility of utilizing free software for processing and manipulating digital dental models.

Materials and methods: The study employed a meticulous approach, utilizing specialized software tools to execute the photogrammetry process. Impressions of the maxillary arch were obtained from 50 subjects, and GM were meticulously crafted from these impressions. The subsequent step involved capturing detailed photographs of each GM from various angles, which were then processed using 3DF Zephyr Free^®^ software by 3Dflow (Verona, Italy). This software, renowned for its semi-automatic functionality, facilitated the reconstruction process by seamlessly processing the uploaded photos. For the measurements, Blender^® ^(Blender Institute B.V., Amsterdam, The Netherlands) - a sophisticated software in digital modeling and animation - was used. While digital calipers were employed to measure the GM, Blender software was utilized to measure the DM.

Results: The results of the analysis, as evaluated by unpaired t-tests and volumetric assessments, revealed no significant discrepancies between the measurements obtained from the GM and the DM generated via photogrammetry.

Conclusion: The findings underscore the efficacy of these software tools in ensuring accuracy and reliability in the digitization process.

## Introduction

In the final decades of the previous century, advancements in computing and the internet brought about a significant transformation in manufacturing, marking the onset of the Third Industrial Revolution. This transformative period paved the way for what we now recognize as the Fourth Industrial Revolution, characterized by the fusion of the physical and digital realms, leading to the development of cyber-physical systems. Unlike its predecessors, this revolution extends its influence beyond large-scale production, impacting various professions by enhancing existing processes and introducing novel technologies that bridge the gap between physical and digital realms. Consequently, traditional techniques are gradually being replaced by faster, more precise, and standardized methods across various domains [1-4].

In the field of dentistry, this evolution is evident in the growing adoption of digital approaches for diagnosis, treatment planning, and execution [5-7]. Patient data, including photographs, radiographs, and dental models, are no longer confined to physical storage but can instead be stored electronically, thereby optimizing space in dental offices and enabling quick and intuitive access to information [1,8].

Choosing the appropriate technique depends on specific circumstances. For instance, intraoral scanners may not be suitable for patients without teeth in their mouths. Therefore, it is crucial to thoroughly assess each situation to identify the most suitable approach [9-12].

Many research studies have been undertaken to compare linear measurements derived from digital dental models with those from traditional plaster models, consistently indicating statistical similarity between the two [13]. Nevertheless, a notable aspect is that many of these studies did not incorporate photogrammetry as a source for acquiring digital dental models.

Photogrammetry is defined by the American Society of Photogrammetry and Remote Sensing as the “art, science and technology of obtaining reliable information about physical objects and the environment, through the process of recording, measuring and interpreting imagery and digital representations of energy patterns derived from non-contact sensor system” [14]. Photogrammetry has shown significant promise in transforming small objects into highly accurate 3D models, faithfully preserving their original size and shape. This technology holds particular potential in dentistry and maxillofacial fields, offering a viable method for generating digital dental models from traditional plaster ones. Moreover, photogrammetry presents a cost-effective alternative to conventional scanning methods, making it an attractive option for digitizing dental models [15].

The objective of this research was to assess the precision and accuracy of creating a digital model (DM) from a gypsum model (GM) through photogrammetry. Furthermore, the study aimed to determine the reliability and precision of photogrammetry as a cost-effective alternative for digitizing GMs, making it a more feasible option for dentists transitioning to digital workflows.

## Materials and methods

Study design

This cross-sectional comparative study was conducted to evaluate the accuracy and reliability of DMs generated using photogrammetry compared to traditional GMs. The study was conducted in two main environments.

Clinical Setting

Patients were randomly selected from the OPD of the department, where alginate impressions were made, and GMs were created.

Laboratory Setting

GMs were measured with a digital caliper, while 3D models were generated using 3DF Zephyr® (3Dflow, Verona, Italy) and analyzed in Blender® software (Blender Institute B.V., Amsterdam, The Netherlands). Fifty dentulous individuals were chosen for the study. Using irreversible hydrocolloid impression material, commonly known as alginate (Tropicalgin, Zhermack, Boca Raton, Florida, USA), separate impressions of maxillary arches were made consecutively at the same visit. The impressions were poured immediately with dental stone (Kalstone, Kalabhai GmbH, Gelnhausen, Germany). The models were later photographed using a digital single-lens reflex camera (Sony Alpha 7 IV, Tokyo, Japan). The study was conducted in the Department of Prosthodontics for six months; data collection for three months, data analysis for one month, and report writing for two months, a total duration was from February 2024 to July 2024. The current study was approved by the Institutional Ethical Committee with approval number: SDCRI/IEC/22/33. Informed consent was taken before the commencement of the study.

Statistical analysis

Descriptive statistics, including mean and standard deviation, were used to report the deviations in key anatomical points. The sample size was calculated based on an effect size of 0.67, derived from a similar study by Stuani et al., which evaluated the accuracy of photogrammetry-generated DMs compared to traditional GMs [1]. Using G Power software (version 3.0; Heinrich-Heine-Universität Düsseldorf, Düsseldorf, Germany), a minimum sample size of 50 was found to be sufficient to achieve a power of 80% with an alpha of 0.05.

Methodology

The inclusion criteria were class-1 facial profile, erupted permanent dentition (first molar to first molar), no missing teeth (first molar to first molar), stable centric occlusion, no pre-dental treatment (orthodontic/restorative), no blebs or voids in the plaster or DMs, and no fractures of the teeth on the plaster models.

Exclusion criteria were patients with a previous history of orthodontic treatment, improper occlusion, fractured tooth/teeth, and mutilated teeth.

Following the inclusion and exclusion criteria, separate impressions of maxillary arches were made consecutively at the same visit for 50 dentulous individuals using irreversible hydrocolloid impression material, commonly known as alginate. These impressions were carefully poured with dental plaster and dental stone to produce precise and detailed GMs, accurately capturing the anatomical features of the maxillary arches.

Following the creation of the GM, the focus shifted to generating digital representations of these models through a photographic method. Each model underwent a thorough photographic process, with 50 photographs taken from various angles under ambient light conditions. All the study models were photographed using a camera mounted on a stand with a macro portrait lens (EF 100mm f/2.8, 1:1 OS, Macro SAL100M28) with automatic settings. These photographs were meticulously captured to ensure comprehensive coverage of the surface anatomy and details of the GM.

**Figure 1:**
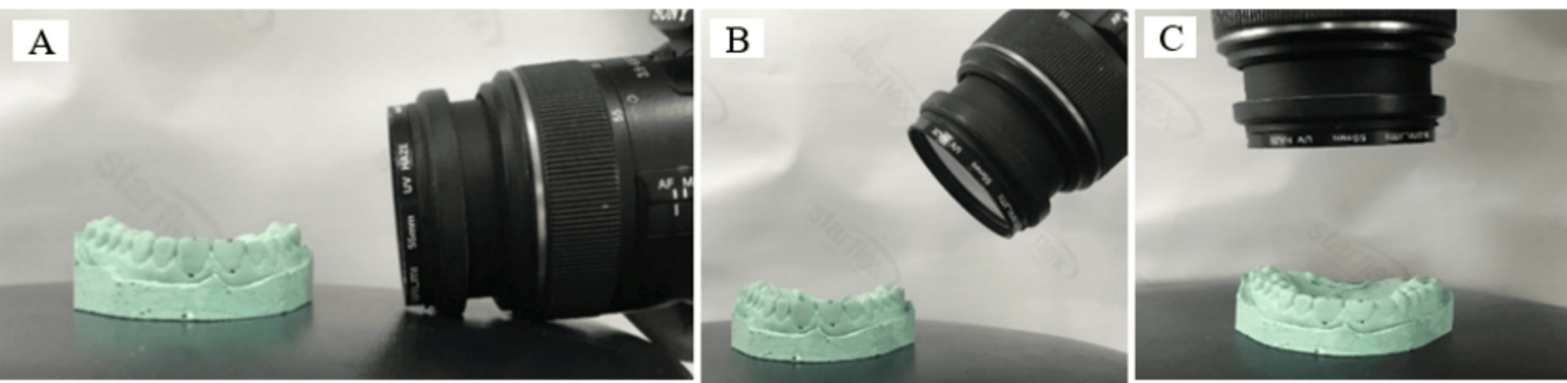
Three perspectives of capturing the photographs

The photographs were captured from three different perspectives:

(1) Twenty-four photos were taken parallel to the occlusal plane of the GM, with one photo captured every 15 degrees (Figure 1A).

(2) Another set of 24 photos was angled at 45 degrees relative to the occlusal plane of the GM, also with one photo taken at every 15-degree interval (Figure 1B).

(3) Finally, two photos were captured perpendicular to the occlusal plane of the GM, with each focusing on one hemi-arch (Figure 1C).

All photographs were captured using a Sony Alpha 7 IV with a macro portrait lens (EF 100mm f/2.8 1:1 OS Macro SAL100M28) set to automatic mode, with ISO 100, aperture f/2.8, and shutter speed 1/160 seconds.

**Figure 2:**
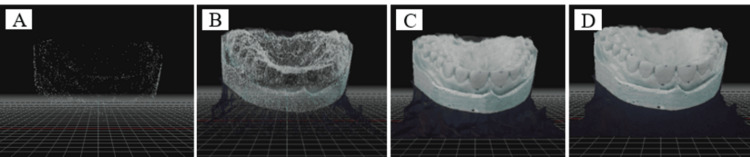
Producing models using 3DF Zephyr® software

The pictures acquired from the photographic procedure underwent processing through the 3DF Zephyr software. This software streamlines the reconstruction process by providing a semi-automatic approach, enabling users to upload photos and adjust reconstruction settings with ease (Table 1 and Figure 2).

**Table 1:** Workflow of the software used to generate the photographs of the study models

Workflow of the software	
Uploading the picture	Each set was opened as a new project, and all corresponding photos were uploaded.
Employing the software	The "Masquerade" plugin within the software was employed to mask the background in the photos.
Generating the photos	A sparse point cloud was generated by pairing the photos, with the settings configured to "Close Range" and "Deep" (Figure 2A).
	Subsequently, a dense point cloud was created, utilizing the settings "Close Range" and "High Details" (Figure 2B).
	A mesh was then extracted from the dense point cloud, using the above settings (Figure 2C).
	Following this, a textured mesh was generated by adjusting the settings to "General" and "Default Single Texture" (Figure 2D).
	Finally, the textured mesh was exported from the software in OBJ/MTL format.

After the DMs were generated, specific measurements were taken to analyze the anatomical features of the maxillary arches. The height of both central incisors and the distances between the canine (C) cusps (C-C) and between the mesial marginal ridge of the first premolar (1PM) and distal marginal ridge of the first molar (1M) (1PM-1M) on each side of the arch were recorded.

For recording the measurements in traditional GMs, measurements were made using a digital caliper with 0.01 mm sensitivity. In contrast, the DMs were measured using Blender software with the analysis tools provided to the nearest 0.1 mm, which offered precise measurement capabilities.

In the Blender software, measurements were conducted with the height of the central incisor serving as the reference point (Figures 1B, 2C). This approach ensured consistency and accuracy in the measurement process across all DMs generated from the photographs. Using the central incisor's height as a reference, it allowed for precise comparisons and analysis of the anatomical features of the maxillary arches between the traditional GM and the DM.

Statistical testing

Statistical analysis was performed using the Statistical Package for the Social Sciences (SPSS) version 22.0 (IBM® SPSS® Armonk, NY, USA). Descriptive statistics, including mean values and standard deviations, were calculated for all measurement parameters. To assess the normality of the data, the Shapiro-Wilk test was employed, which is appropriate for small to medium sample sizes. The results indicated that the data were normally distributed (p > 0.05). Consequently, parametric tests, specifically the unpaired t-test, were used to compare the accuracy of DMs generated using photogrammetry with traditional GMs. Additionally, Levene's test for equality of variances was applied to ensure that the variances were equal across the groups, confirming the appropriateness of the t-test for further comparisons.

Normality of data

The normality of the data was assessed using the Shapiro-Wilk test, which is widely used in dental research to verify normal distribution for continuous variables. The test results indicated that the data followed a normal distribution (p > 0.05), allowing for the application of parametric statistical tests. This justifies the use of unpaired t-tests for comparing the measurements obtained from digital and traditional GMs. These tests were chosen due to their sensitivity in detecting differences in means between two groups when data is normally distributed.

## Results

In the comparative analysis between DMs generated using photogrammetry and traditional GMs for 50 dentulous subjects, unpaired t-tests were utilized to assess the mean values across various dental parameters. The results revealed no statistically significant differences between DM and GM measurements for the key parameters.

C-C distance

The mean distance between the C-C was compared using an unpaired t-test. The statistical outcome indicated no significant difference between the DM and GM groups, with a t-value of 0.649 and a p-value of 0.518. This suggests that both methods provided comparable accuracy in measuring the C-C distance (Table 2).

**Table 2:** Comparison of mean canine to canine distance (in mm) between digital models (DMs) and gypsum models (GMs)

Models	Minimum (in mm)	Maximum (in mm)	Mean	Std. deviation	t-value	p-value
GM	29.4	38.9	34.18	2.05	0.649	0.518
DM	29.4	39.5	34.44	2.05		

1PM-1M (first quadrant) distance

The comparison of the distance between the mesial marginal ridge of the 1PM and the distal marginal ridge of the 1M in the first quadrant resulted in a t-value of 0.395 and a p-value of 0.694, indicating no significant disparity between the two groups (Table 3).

**Table 3:** Comparison of mean first premolar-first molar (first quadrant) distance (in mm) between digital models (DMs) and gypsum models (GMs)

Models	Minimum (in mm)	Maximum (in mm)	Mean	Std. deviation	t-value	p-value
GM	20.8	29.2	23.73	1.29	0.395	0.694
DM	21.0	29.2	23.83	1.29		

1PM-1M (second quadrant) distance

For the 1PM-1M distance in the second quadrant, the analysis showed a t-value of 0.951 and a p-value of 0.344. These findings further indicate that there was no significant difference between DM and GM measurements for this parameter (Table 4).

**Table 4:** Comparison of mean first premolar-first molar (second quadrant) distance (in mm) between digital models (DMs) and gypsum models (GMs)

Models	Minimum (in mm)	Maximum (in mm)	Mean	Std. deviation	t-value	p-value
GM	20.7	28.2	23.65	1.38	0.951	0.344
DM	20.9	28.7	23.93	1.55		

Overall volumetric comparison

A volumetric assessment was also conducted to compare the total volume of DM and GM. The comparison yielded a p-value of 0.605, indicating no significant differences in volume between the two methods. This result confirms the volumetric accuracy of DM when compared to GM.

Statistical significance of parameters

Across all parameters, the p-values reported exceeded the 0.05 threshold, suggesting that there were no significant differences between the DMs generated using photogrammetry and the traditional GMs. The consistency in measurements across all parameters highlights the reliability of photogrammetry as a method for generating accurate DMs.

Overall, these findings suggest a strong alignment between the measurements obtained from digital and GMs, indicating that photogrammetry-based DMs are as reliable and accurate as traditional GMs for dental measurements.

## Discussion

This study delved into the comparative assessment of the reliability and efficacy of DM versus GM in prosthodontic applications, aiming to elucidate any significant differences in measurements obtained from these two modeling methods.

Plaster of Paris (PoP) and dental stone were selected for the study due to their established usage and suitability for creating DMs. While PoP has been traditionally favored for its ability to accurately capture fine details and its cost-effectiveness, the dental stone was also utilized when the clarity of the 3D models produced with PoP was insufficient. This decision was made to ensure that the resulting models met the required standards of clarity and accuracy for the study.

The software 3DF Zephyr and Blender were chosen for the study as they were available for free and provided the necessary functionalities for processing and manipulating the DM. The 3DF Zephyr software was utilized in the study for its robust photogrammetry capabilities. This software allowed for the creation of accurate 3D models from sets of photographs, which was essential for digitizing the dental impressions. Leveraging the features of 3DF Zephyr enabled the study to efficiently process the acquired data and generate high-quality DMs. Additionally, being available for free, this software provided a cost-effective solution for the research endeavor as suggested in the previous studies [16,17].

On the other hand, Blender software was utilized for measurements in the study due to its comprehensive set of tools for analyzing and quantifying various aspects of the DM. Its versatile measurement features allowed for precise and accurate assessments of dental parameters such as distances, angles, and dimensions. By employing Blender for measurements, the study could ensure consistency and reliability in the analysis of the DMs, thereby facilitating the comparison between different materials and techniques. Additionally, Blender's compatibility with other software used in the study, such as 3DF Zephyr, facilitated seamless data integration and workflow management. Overall, Blender proved to be a valuable tool for conducting measurements and quantitative analyses in the study, contributing to the robustness and rigor of the research findings.

The findings unveiled a notable consistency between DM and GM measurements across the parameters scrutinized in this study. Remarkably, the C-C distance, 1PM-1M (first quadrant) distance, and 1PM-1M (second quadrant) distance showed no statistically significant variances between the two modeling techniques. These results underscore the comparable accuracy and reliability of both DM and GM in capturing dental dimensions critical for prosthodontic treatment planning. Zotti et al. in their study indicated that photogrammetry is a reliable method for generating DMs from plaster models [8].

These outcomes resonate with previous research in prosthodontics, which has consistently demonstrated the efficacy of DM in various clinical scenarios. Photogrammetry, in particular, has emerged as a reliable technique for generating accurate 3D models in prosthodontic applications. This technique utilizes photographs of dental arches taken from multiple angles to reconstruct precise 3D models, offering advantages such as non-invasiveness, cost-effectiveness, and high accuracy [1].

Similarly, Ma et al. compared the accuracy of photogrammetry, intraoral scanning, and conventional impression techniques for complete-arch implant rehabilitation. Their results revealed that photogrammetry exhibited the highest accuracy among the evaluated impression techniques, followed by conventional impressions, while intraoral scanning showed the least accuracy [18].

Possible disadvantages of using photogrammetry in replicating the models are lower accuracy since it is influenced by many factors, such as sensor size, aperture, resolution, and focal length.

Clinical significance

Photogrammetry has several promising clinical implications in dentistry, particularly in areas like storage, model analysis, and treatment planning. Here are some key ways it can impact dental practice:

Digital Storage and Management

Efficient storage: Photogrammetry enables the creation of high-resolution 3D models of dental structures, which can be stored digitally eliminating the need for physical plaster models, reducing storage space and the risk of damage or loss.

Cloud-based accessibility: DMs can be stored in cloud systems, allowing for easy access and sharing among practitioners, patients, and labs, thus improving collaboration and long-term record-keeping.

Study and Analysis of Models

Precise measurements: 3D models generated through photogrammetry allow for highly accurate measurements of dental structures, which can be critical in assessing tooth positions, occlusions, and soft-tissue anatomy.

Virtual simulations: Dentists can manipulate the DMs in various ways, including rotating, zooming, and sectioning, to study the patient's anatomy in more detail. This is especially useful for educational purposes or when analyzing complex cases.

Pre-treatment analysis: By comparing pre-treatment and post-treatment models, practitioners can assess the progress of treatment more effectively and make informed decisions about adjustments.

Treatment Planning

Orthodontic applications: 3D models can be integrated into computer-aided design/computer-aided manufacturing (CAD/CAM) software for custom treatment planning in orthodontics, such as planning for braces or clear aligners. The high accuracy of photogrammetry models ensures better fitting appliances.

Implant planning and surgery: Photogrammetry provides highly detailed 3D reconstructions of the dental arches, helping to accurately plan implant placement. The models allow for a detailed evaluation of bone structures, which is crucial for implant success.

Restorative dentistry: Precise 3D models can be used for the design of crowns, bridges, and other restorations, ensuring a perfect fit. By integrating these models with 3D printers or milling machines, custom restorations can be produced with minimal error.

Patient Communication

Visualization for patients: Having 3D models allows patients to better understand their dental condition and the proposed treatment plans. Visual representation helps in enhancing patient acceptance of complex treatment plans.

Simulation of outcomes: Dentists can simulate post-treatment results, like how the teeth would look after orthodontic treatment or after implants, providing a clear expectation for the patient.

Minimally Invasive Procedures

Pre-surgical planning: Using photogrammetry in conjunction with other digital tools allows for detailed pre-surgical planning, potentially reducing surgery time and increasing precision.

Guided surgery: The 3D models can be used to create surgical guides for implants or other procedures, leading to more accurate and minimally invasive treatments.

Cost and Time Efficiency

Reduced costs: Once the initial equipment is set up, using photogrammetry can be more cost-effective than traditional impressions and models, as there is no need for impression materials or physical storage.

Time-saving: Digital workflows enabled by photogrammetry reduce the time needed to make adjustments, re-take impressions, or produce new physical models, streamlining the overall treatment process.

Limitations

The relatively limited sample size might restrict the applicability of the findings to broader patient cohorts. The study focused on a subset of dental parameters, neglecting factors such as material properties and fabrication techniques. A limitation arose as Blender software was unable to replicate models with the same clarity achieved in 3DF Zephyr.

## Conclusions

This study contributes valuable insights into the growing body of evidence supporting the integration of DMs, particularly those generated through photogrammetry, in prosthodontic practice. The findings underscore the potential of DMs as a reliable alternative to traditional GMs, offering advantages such as enhanced storage, accessibility, and ease of manipulation. Prosthodontic clinicians can confidently incorporate photogrammetry-derived DMs into their clinical workflow, leveraging their accuracy and reliability for treatment planning and prosthesis fabrication. Subsequent studies could delve further into these aspects to offer a more comprehensive insight into the comparative effectiveness of digital and GMs in various prosthodontic applications.
